# Neural‐hematopoietic‐inflammatory axis in nonsmokers, electronic cigarette users, and tobacco smokers

**DOI:** 10.14814/phy2.15412

**Published:** 2022-10-05

**Authors:** Isabelle Ruedisueli, Sara Arastoo, Pawan K. Gupta, Jeffrey Gornbein, Holly R. Middlekauff

**Affiliations:** ^1^ Division of Cardiology, Department of Medicine David Geffen School of Medicine at UCLA Los Angeles California USA; ^2^ Department of Anesthesiology and Perioperative Medicine David Geffen School of Medicine at UCLA Los Angeles California USA; ^3^ Department of Molecular and Medical Pharmacology David Geffen School of Medicine at UCLA Los Angeles California USA; ^4^ Departments of Medicine and Computational Medicine David Geffen School of Medicine at UCLA Los Angeles California USA

## Abstract

Amygdala activity in context of the splenocardiac model has not been investigated in healthy, young adults and has not been compared between nonsmokers, electronic cigarette users, and smokers. The purpose of the current study was to determine whether fluorodeoxyglucose positron emission tomography/computer tomography (FDG PET/CT) scans would demonstrate positively correlated metabolic activity in the amygdala, bone marrow, spleen, and aorta, elucidating activation of the splenocardiac axis in otherwise healthy young people who use tobacco products compared to nonusers. Moreover, the study was conducted to evaluate whether electronic cigarette users and tobacco smokers have similar levels of inflammation compared to nonusers. In 45 healthy adults (mean age = 25 years), including nonsmoker (*n* = 15), electronic cigarette user (*n* = 16), and smoker (*n* = 14) groups, metabolic activity in the amygdala, spleen, aorta, bone marrow of thoracic vertebrae, and adjacent erector spinae skeletal muscle was quantified through visualization of radioactive glucose (^18^FDG) uptake by FDG‐PET/CT. The maximum standardized uptake value for each region was calculated for correlation analyses and comparisons between groups. In correlation analyses, metabolic activity of the amygdala correlated with metabolic activity in the aorta (*r* = 0.757), bone marrow (*r* = 0.750), and spleen (*r* = 0.665), respectively. Metabolic activity in the aorta correlated with ^18^FDG uptake in the thoracic vertebrae (*r* = 0.703) and spleen (*r* = 0.594), respectively. Metabolic activity in the spleen also correlated with ^18^FDG uptake in the bone marrow (*r* = 0.620). Metabolic activity in the adjacent erector spinae skeletal muscle (our control tissue) was not positively correlated with any other region of interest. Finally, there were no statistically significant mean differences in metabolic activity between the three groups: nonsmokers, electronic cigarette users, and smokers in any target tissue. Amygdala metabolic activity, as measured by ^18^FDG uptake in FDG‐PET/CT scans, positively correlated with inflammation in the splenocardiac tissues, including: the aorta, bone marrow, and spleen, underscoring the existence of a neural‐hematopoietic‐inflammatory axis in healthy, young adults.

## Graphical abstract

To our knowledge, this is the first study to probe for the network of the splenocardiac axis in healthy young people, including a cohort who uses tobacco products. We found moderate‐strong correlation between metabolic activity in all regions of interest, including the amygdala, bone marrow, spleen, and aorta, but not control tissue, skeletal muscle. There were no differences in metabolic activity in any target tissue between cohorts. Our findings are consistent with the early establishment of this network of inflammation in otherwise healthy young adults.

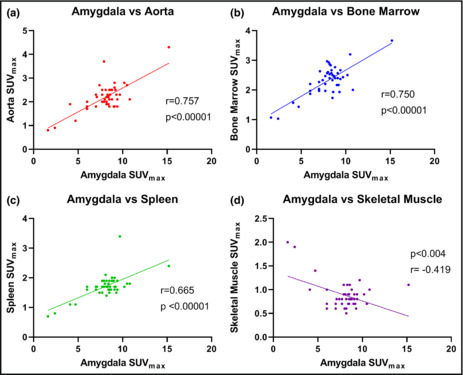

## 1 INTRODUCTION

The lipid‐centric view of atherosclerosis, as a disease resulting from lipid accumulation in the atheromata, has been upended by growing research into the effects of systemic inflammation (Libby & Hansson, 2019). This shift is best embodied by the proposed developmental model of atherosclerosis and acute myocardial infarction: the splenocardiac axis (Libby et al., 2016). The splenocardiac axis effectively connects the sympathetic nervous system, bone marrow, spleen, and aorta in the genesis of inflammation (Libby et al., 2016). During stress, increased sympathetic tone activates bone marrow progenitor cells and leukocytes via B‐3 adrenergic stimulation (Dutta et al., 2012; Laukova et al., 2012; Libby et al., 2016). These progenitor cells then voyage to the spleen, where they proliferate due to stem cell factors (Dutta et al., 2012). Elevated numbers of proinflammatory monocytes finally transit to the arterial wall, recruited by nascent atherosclerotic plaques and aggravate the development of atherosclerosis (Dutta et al., 2012; Laukova et al., 2012; Libby et al., 2016).

The splenocardiac axis may elucidate the experience of stress, physiologically manifested through increased sympathetic tone, as the origin of atherosclerotic development (Libby et al., 2016). Measurement of target tissues in the splenocardiac axis has been largely performed through ^18^F‐fluorodeoxyglucose positron emission tomography/computer tomography (FDG‐PET/CT) (Boas et al., 2017; Rudd et al., 2007; Tahara et al., 2006; Tawakol et al., 2013). Activated inflammatory cells express high levels of glucose transporters; FDG‐PET/CT detects glucose uptake and inflammation in target tissues (Maratou et al., 2007). Prior research utilizing FDG‐PET/CT has imaged portions of the axis; increased splenic activity correlated with arterial inflammation and independently predicted risk of cardiovascular disease events (Emami et al., 2015). However, complete analysis of the splenocardiac model which is instigated by increased sympathetic tone, warrants inclusion of its neural origin: the amygdala.

The amygdala receives input from the brainstem and coordinates activity of the midbrain dopaminergic neurons, moderating behavioral and autonomic responses to emotional stimuli (Fudge & Emiliano, 2003; Ledoux et al., 1988; Steinberg & Janak, 2013; Veening et al., 1984). When activated by fear or stress, the amygdala sends action potentials down efferent neurons that project to the brainstem and instigate a sympathetic response (Ledoux et al., 1988; Rodrigues et al., 2009). As an initiator of sympathetic nerve activity, the amygdala embeds itself in discussions of the splenocardiac axis; prior research has illuminated this connection (Macefield et al., 2013). Heightened amygdala activity, due to psychosocial stress, has been shown to predispose individuals to development of atherosclerosis (Powell‐Wiley et al., 2021). Amygdala activity has also independently predicted cardiovascular disease events, as mediated by increased bone marrow activity and arterial inflammation (Tawakol et al., 2017). Therefore, a holistic investigation of the splenocardiac axis mandates inclusion of the amygdala.

Tobacco cigarette smoking currently stands as the leading modifiable cause of heart disease‐related death in the United States, totaling >480,000 deaths per year (Lariscy, 2019; Warren et al., 2014). Tobacco cigarettes increase central sympathetic outflow as well as catecholamine release from adrenergic terminals (Grassi et al., 1992). Nicotine, the addictive constituent of tobacco cigarettes, acts on nicotinic acetylcholine receptors present in the central nervous system, autonomic ganglia, and neuromuscular junction (Brunzell et al., 2015; Lee et al., 2019). Exposure to nicotine acutely increases heart rate and blood pressure, indicating sympathetic dominance (Benowitz, 1997). Elevated circulating white blood cells have also been noted in chronic smokers compared to nonsmokers, suggesting a low‐grade inflammatory response in the presence of habitual smoking (Higuchi et al., 2016). However, the effects of chronic tobacco product use, including tobacco cigarettes and electronic cigarettes on the *amygdala* have not been studied and remain poorly understood.

Prior research has demonstrated that nicotine's presence may encourage synaptic plasticity in the amygdala while its absence may provoke amygdala activation during withdrawal periods (Brody et al., 2002; Huang et al., 2008; Wang et al., 2007). Nicotine binds to dopaminergic neurons in the ventral tegmental area which project, in part, to the amygdala (Brody et al., 2002; Kalivas & Volkow, 2005; Salamone John & Correa, 2012; Volkow et al., 2019). Chronic exposure to nicotine was shown to create long term synaptic potentiation or long‐lasting modifications of synapses in the amygdala (Huang et al., 2008). Additionally, withdrawal symptoms, occurring as early as 4 h after smoking cessation, have been positively associated with amygdala activation and modifications in anatomically and functionally connected neural regions (Craig, 2009; Critchley, 2009; Ghahremani et al., 2021; Hughes, 2007; Perez Diaz et al., 2021; Wang et al., 2007). In separate investigations, chronic smokers who abstained for 12 h demonstrated increased activity in the amygdala, distinct functional connectivity patterns of the anterior insula with the anterior cingulate cortex, and decreased right anterior insula thickness as correlated with intensity of craving (Ghahremani et al., 2021; Perez Diaz et al., 2021; Wang et al., 2007). Aforementioned investigations suggest an association between abstinence‐induced craving and changes in affective and cognitive regions (Ghahremani et al., 2021; Perez Diaz et al., 2021; Wang et al., 2007). Lastly, the c‐fos gene, a marker for neuronal activation, was activated in the amygdala during acute nicotine withdrawal in rats, garnering further support for the amygdala's intimate relationship with nicotine (Panagis et al., 2000).

To this end, the ebb and flow of nicotine exposure in habitual smokers, exemplified in smoking and the subsequent onset of acute withdrawal symptoms, may lead to more frequent sympathetic nerve activation as a result of amygdala arousal. Analyzing morning amygdala activity in chronic users before daily smoking initiation could potentially uncover increased metabolic activity in this region due to the acute onset of withdrawal. This activity, as elucidated by the splenocardiac model, may put these individuals at greater risk for the downstream development of atherosclerosis compared to nonsmokers. As previously mentioned, there is burgeoning literature associating amygdala activation with inflammation in the target tissues of the splenocardiac axis (Boas et al., 2017; Powell‐Wiley et al., 2021; Tawakol et al., 2017). With this, we hypothesized that FDG‐PET/CT scans would demonstrate positively correlated metabolic activity in the amygdala, bone marrow, spleen, and aorta, elucidating activation of the splenocardiac axis in otherwise healthy young people who use tobacco products compared to nonusers. We also hypothesized that inflammation would be similar in smokers and electronic (e) cigarette users, who are similarly nicotine‐addicted compared to nonsmokers.

## 2 MATERIALS AND METHODS

### 2.1 Study population

Otherwise healthy smokers, e‐cigarette users, and nonsmokers between the ages of 21–45 years were recruited. Eligible smokers and e‐cigarette users included individuals who had smoked cigarettes or e‐cigarettes, respectively, for >1 year prior to enrollment. Dual users did not qualify. Nonsmokers included individuals who did not smoke or use e‐cigarettes, although former tobacco smokers who had stopped smoking for >1 year prior to enrollment were eligible. In addition to meeting the aforementioned smoking criteria, individuals from all groups were enrolled if they had no known health problems, were nonobese (<30 kg/m^2^ BMI), were not taking prescription medications (excluding oral contraceptives), had an alcoholic intake <2 drinks/day and no illicit drug use (by self‐report, and confirmed by urine toxicology testing), were not pregnant (urine pregnancy test administered on the day of study), and had not been exposed to secondhand smoke and were not using nicotine replacement therapies. To balance age and sex between groups, participants were enrolled accordingly. The experimental protocol was approved by the Institutional Review Board at the University of California, Los Angeles and written informed consent was obtained from each participant. H.R.M. had full access to all the data in the study and takes responsibility for its integrity and the data analysis.

### 2.2 FDG‐PET/CT imaging and venipuncture

Following previously devised standards and guidelines, dedicated research FDG‐PET/CT imaging was performed to ensure maximum FDG uptake in the amygdala, hematopoietic tissues, and arterial wall (Boas et al., 2017). After an overnight fast and confirmation of fasting blood glucose <100 mg/dl, 0.14 mCi/kg of ^18^F‐FDG was injected intravenously. The subject rested for 90‐min post injection and then images of the head, neck, chest, and abdomen were obtained. High count 5‐min scans per bed position were taken in place of the shorter, 2‐min scans conventionally performed for oncology imaging. This approach produced a higher count rate and decreased image noise, leading to better image quality for image analysis. Blood samples were retrieved through venipuncture by trained Nuclear Medicine staff on the day of the study. Samples were sent to the UCLA Clinical Laboratory for cotinine measurement (cotinine *t*_1/2_ 20 h).

### 2.3 Image analysis

Scans were read by a single investigator (P. K. G.) blinded to study group affiliation and study outcomes. As noted in prior reporting, metabolic activity of the amygdala, aorta, spleen, thoracic vertebrae, and adjacent erector spinae skeletal muscle were measured by placing a region of interest over axial sections (Bucerius et al., 2014; Emami & Tawakol, 2014; Tawakol et al., 2013). The maximum standardized uptake value (SUV_max_) of ^18^F‐FDG was recorded for each region (Chen & Dilsizian, 2015; Huet et al., 2015). The amygdala was identified along the medial temporal lobe and the volumetric region of interest was drawn based on localization using multi‐format image reconstruction. The SUV_max_ and mean standardized uptake value (SUV_mean_) were calculated for left and right amygdalae. Furthermore, analysis of the aorta involved SUV_max_ measurements taken every 5 mm, starting at 1 cm above the aortic valve annulus until the bottom of the aortic arc (Goyal et al., 2020; Tawakol et al., 2017). Finally, for all other outcomes, including the spleen, bone marrow (average of three thoracic vertebrae, T9, T10, T11), and skeletal muscle control, the SUV_max_ was measured in the axial plane.

### 2.4 Sample size calculation

A sample size of *n* = 45 allows confirmation of correlations as small as *r* = 0.40 with 80% power and using the *α* = 0.05 significance criterion. Our lab previously investigated correlations between cotinine hematopoietic tissue metabolic activity, where only correlations larger than *r* = 0.39 were clinically relevant (Boas et al., 2017).

### 2.5 Statistical analysis

The *p* values for comparing continuous variables among the three groups were computed using a parametric one way analysis of variance (ANOVA) model if the continuous variable had a normal distribution or was computed using the non‐parametric Kruskal‐Wallis method otherwise. The *p* values for comparing categorical variables such as sex among the three groups were computed using Fisher's exact test. No adjustment was made for multiple comparisons across variables. The Fisher LSD criterion was used to control for multiple comparisons for a given variable. The Shapiro‐Wilkes statistic was computed using the residual errors to help determine if each outcome had a normal distribution.

Pearson correlations were computed for assessing correlations among amygdala variables, among aortic variables or between amygdala versus aortic variables. Correlations were computed for all three groups combined controlling for possible group effects by centering. Plots are provided with the corresponding linear regression line.

## 3 RESULTS

### 3.1 Study population

A total of 45 individuals were enrolled in the study, including 14 smokers, 16 e‐cigarette users, and 15 nonsmokers. Overall sample characteristics and group‐divided characteristics (tobacco smokers, e‐cigarette users, and nonsmokers) are displayed in Table 1. There were no significant differences between groups for any descriptive statistics. Plasma cotinine levels were non‐detectable (<2 ng/ml) in all of our nonsmokers, consistent with their non‐smoking status. Cotinine levels were not significantly different between e‐cigarette users (80.0 ng/ml) and smokers (85.0 ng/ml), and were relatively low, consistent with light tobacco product use in our cohort (Table 1).

**TABLE 1:** Study population characteristics

	Nonusers	E‐cigarette users	Smokers	*p*‐value	Total
Sample size (*n*=)	15	16	14		45
Age	25.1 ± 4.3	25.1 ± 4.0	26.8 ± 5.8	0.6438	25.6 ± 4.7
Sex (F/M)	6/9	6/10	7/7	0.8056	19/26
BMI (kg/m^2^)	22.9 ± 2.7	23.6 ± 3.3	22.9 ± 2.9	0.7347	23.1 ± 2.9
Highest level of education					
College	15	14	13		3
No college	0	2	1	0.6364	42
Cotinine (ng/ml)^a^	<2	80 (50.0–127.5)	85 (17.5–126.3)	0.9990^b^	

*Note*: Values are given as mean ± SD.

Abbreviation: BMI, body mass index.

^a^ Values are given as median (Q1–Q3).

^b^ E‐cigarette users versus smokers.

### 3.2 Mean FDG uptake measure by SUV_max_ in each tissue

Mean FDG uptake for total group (*n* = 45) per tissue of interest was measured (Table 2). FDG uptake was measured in the tissues that compose the splenocardiac axis, including the amygdala, aorta, bone marrow of three thoracic vertebrae (T9, T10, T11) and spleen, and in the skeletal muscle, not part of the splenocardiac axis, thus control tissue. Mean SUV_max_ measurements did not differ significantly between groups (nonsmoker vs. e‐cigarette users vs. smokers) (Table 2). Results were unchanged when amygdala activity was calculated in the left or right amygdala, as SUV_max_ or SUV_mean_.

**TABLE 2:** Mean FDG uptake in target tissue by group

	Nonsmokers (*n* = 15)	E‐cigarette users (*n* = 16)	Tobacco smokers (*n* = 14)	*p*‐value	Total (*n* = 45)
Amygdala	7.37 ± 2.67	8.38 ± 1.13	8.34 ± 2.40	0.5558	8.03 ± 2.15
Aorta	2.18 ± 0.71	2.14 ± 0.30	2.24 ± 0.71	0.8439	2.19 ± 0.59
Bone marrow	2.32 ± 0.68	2.48 ± 0.36	2.62 ± 0.57	0.6492	2.47 ± 0.55
Spleen	1.59 ± 0.42	1.79 ± 0.45	1.72 ± 0.29	0.9974	1.70 ± 0.40
Muscle	1.00 ± 0.41	0.83 ± 0.17	0.83 ± 0.24	0.3480	0.88 ± 0.29

*Note*: Values are given as mean ± SD. All values are SUV_max_.

Abbreviation: SUV_max_, maximum standardized uptake value.

### 3.3 Pearson correlations

**FIGURE 1:**
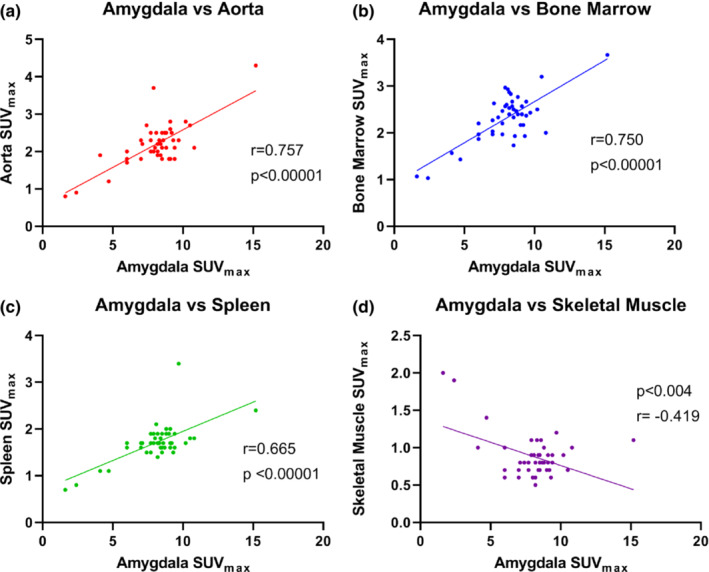
Correlation of metabolic activity in the amygdala with various target tissues: Aorta, bone marrow, spleen, and skeletal muscle control (*n* = 45). Panel (a) FDG uptake in the amygdala positively correlated with FDG uptake in the aorta (*r* = 0.757). Panel (b) FDG uptake in the amygdala positively correlated with FDG uptake in the bone marrow (*r* = 0.750). Panel (c) FDG uptake in the amygdala positively correlated with FDG uptake in the spleen (*r* = 0.655). Panel (d) FDG uptake in the amygdala was not positively correlated, and in fact was weakly negatively correlated with FDG uptake in the skeletal muscle control. FDG, ^18^F‐fluorodeoxyglucose; SUV_max_, standardized uptake value.

**FIGURE 2:**
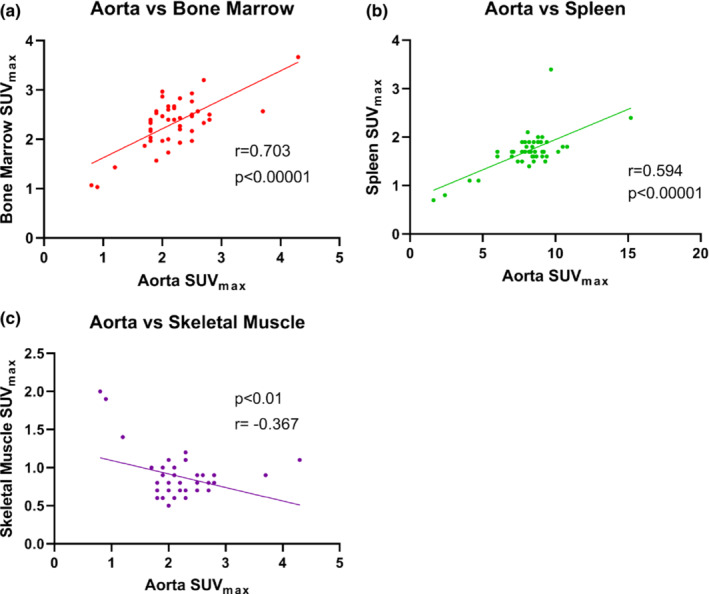
Correlation of metabolic activity in the aorta with various tissues of the splenocardiac axis: Bone marrow, spleen, and skeletal muscle (*n* = 45). Panel (a) FDG uptake in the aorta is positively correlated with FDG uptake in the bone marrow (*r* = 0.703). Panel (b) FDG uptake in the aorta is positively correlated with FDG uptake in the spleen (*r* = 0.594). Panel (c) As expected, FDG uptake in the aorta was not positively correlated, and in fact was weakly negatively correlated with FDG uptake in the skeletal muscle control (*r* = −0.367). FDG, ^18^F‐fluorodeoxyglucose; SUV_max_, standardized uptake value.

**FIGURE 3:**
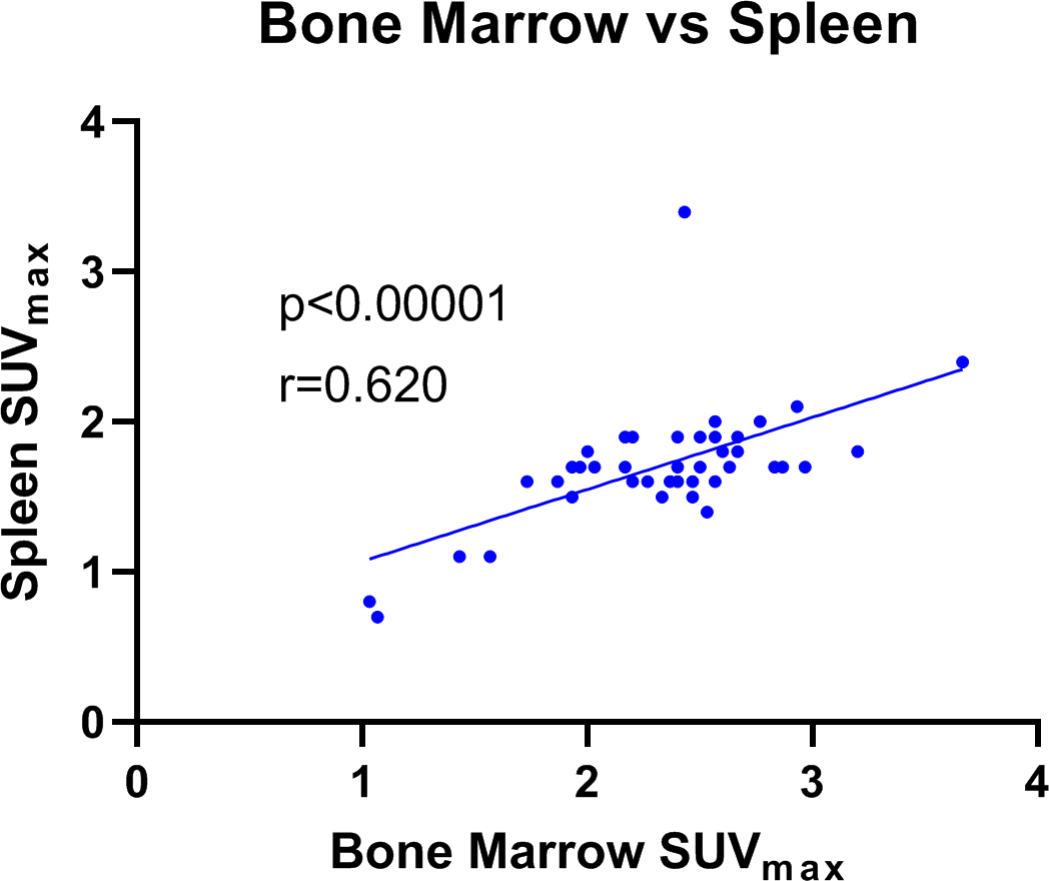
Correlation of metabolic activity in the bone marrow and spleen (*n* = 45). FDG uptake in the bone marrow positively correlated with FDG uptake in the spleen (*r* = 0.621). FDG, ^18^F‐fluorodeoxyglucose; SUV_max_, standardized uptake value.

For the total sample (*n* = 45), there was significant positive correlation in metabolic activity, demonstrated by SUV_max_, in all target tissues in the splenocardiac axis, but not in the extra‐splenocardiac axis tissue, the skeletal muscle (as expected) (Table 3). There was a moderate to strong positive correlation between metabolic activity in the amygdala with metabolic activity in the aorta, bone marrow, and spleen, respectively (Figure 1a–c). There was a weak negative correlation with amygdala metabolic activity and skeletal muscle (control) (Figure 1d). Furthermore, there was moderate to strong correlation between FDG uptake in the aorta and bone marrow as well as the aorta and spleen (Figure 2a,b). Correlation between the spleen and bone marrow was also significant (Figure 3). FDG uptake in skeletal muscle was not positively correlated with any target tissues, as predicted (Figure 3; Table 3).

**TABLE 3:** Pearson correlations controlling for group

	Amygdala	Aorta	Bone marrow	Spleen	Skeletal muscle
Amygdala	**1**	0.757	0.750	0.665	−0.419
Aorta	0.757	**1**	0.703	0.594	−0.367
Bone marrow	0.750	0.703	**1**	0.620	−0.423
Spleen	0.665	0.594	0.620	**1**	−0.269
Skeletal muscle	−0.419	−0.367	−0.423	−0.269	**1**

*Note*: Red text indicates moderate to strong correlation, *p* < 0.01.

Blue text signifies each tissue correaltion with itself.

## 4 DISCUSSION

The accumulation of inflammatory cells, primarily macrophages, laden with lipids in the arterial wall is recognized as the primary driver of atherosclerosis (Bobryshev et al., 2016). Pro‐inflammatory cytokines migrate to the arterial wall and contribute to plaque build‐up, escalating risk of thrombosis, and cardiac ischemia (Libby, 2021). The mechanism of migration has been detailed through the “splenocardiac axis” (Libby et al., 2016). This model asserts that increased sympathetic nervous activity results in heightened progenitor release in the bone marrow, further multiplication of the progenitor cells due to hematopoietic growth factors in the spleen, and transit of these monocytes to the atherosclerotic plaque (Boas et al., 2017; Libby et al., 2016). Sympathetic nerve activation, most commonly due to acute or chronic stress and pain, associates the amygdala with this model and offers an emotional origin for the inflammation‐driven axis (Muscatell et al., 2015). Therefore, elucidating the validity of the axis requires examination of the amygdala as well.

PET/CT imaging allows for a reliable analysis of metabolic activity indicative of active inflammation in tissues, visualized through increased FDG uptake and quantified through standardized uptake values (SUV_max_) (Bucerius et al., 2014; Emami & Tawakol, 2014; Tawakol et al., 2013). Elevated uptake of FDG in cells demonstrates greater metabolic activity, most prominently seen in activated immune cells influenced by pro‐inflammatory cytokines (Wu et al., 2013). Thus, analysis of PET/CT scans by a reliable investigator offers an objective quantification of inflammation in various regions of interest, permitting correlation analysis among these tissues.

Overall, our results display a moderate to strong correlation among all regions of interest: amygdala, bone marrow, spleen, and aorta. These associations underscore the existence of the splenocardiac axis, or more recently termed the neural‐hematopoietic‐inflammatory axis, as the underlying mechanism for the development of plaque accumulation in the arterial wall (Powell‐Wiley et al., 2021). Exemplified in the metabolic relationship between the amygdala and aorta in our study, stress may play an explicit role in the development of cardiovascular disease. Moreover, our hypothesized model was reinforced by the weak and negative correlation between inflammation in the skeletal muscle, our control, and all other regions of interest. Our findings reinforce those of Tawakol and Powell‐Wiley, suggesting that amygdala activation acts as an instigator for hematopoiesis with important implications for atherosclerosis (Powell‐Wiley et al., 2021; Tawakol et al., 2017).

Prior investigations have largely focused on the connection between the development of cardiovascular disease and amygdala activation in middle‐age adults with comorbidities (ex: hypertension, diabetes mellitus, obesity) (Powell‐Wiley et al., 2021; Tawakol et al., 2017). Our study offers new insight into the role of the splenocardiac axis in young, healthy adults. With a median age of 25 years, variables confounding the investigation of inflammation in the arterial wall, outside of e‐cigarette and tobacco cigarette use, are limited. Therefore, the correlation between metabolic activities in all regions of interest in this young sample is remarkable. Our findings may offer greater insight into an inevitable and early‐established network of inflammation, exacerbated over time due to increased sympathetic nervous response to various stressors.

Surprisingly, there were no differences between groups (nonsmokers, e‐cigarette users, smokers) for metabolic activity in each target tissue. Although acute onset of nicotine withdrawal was hypothesized to increase inflammation by the neural‐hematopoietic‐inflammatory axis, tobacco smokers and e‐cigarette users demonstrated similar metabolic activity with nonsmokers in all target tissues (Panagis et al., 2000; Wang et al., 2007). However, this similarity may result from infrequent use of e‐cigarettes and tobacco cigarettes in our participants, as mean cotinine levels for each group were indicative of light e‐cigarette use and cigarette smoking compared to levels recorded for chronic smokers in prior investigations (Rapp et al., 2020; Wall et al., 1988). Therefore, heavier habitual smoking and e‐cigarette use, quantified by mean cotinine level, may more easily uncover inflammatory differences between groups (Raja et al., 2016). Moreover, our results align with Sahota's investigation of the effect of e‐cigarette use on vascular inflammation. Their research also found no measurable differences in inflammation of the arterial wall, measured by PET/CT, between e‐cigarette users, smokers, and nonsmokers (Sahota et al., 2021).

## 5 FUTURE DIRECTIONS

Interestingly, previous research has shown that sudden cardiac risk significantly decreases within a few months or years after smoking cessation (Sandhu et al., 2012; Thun et al., 2013). Therefore, utilizing PET/CT to track inflammatory changes from the start of smoking cessation through its duration may illuminate the implications of smoking on the heart by monitoring its reversal. Studies in former smokers would be of interest. Furthermore, as our study sample is fairly young, it would be worth exploring the effects of long‐term e‐cigarette use versus tobacco cigarette smoking in “longtime” (>15 years) e‐cigarette users and smokers. With increased chronic exposure to each product, tobacco cigarette or e‐cigarette, there may be more pronounced differences in inflammation between groups.

## 6 LIMITATIONS

There are several limitations in our analysis. Firstly, our sample size is small (*n* = 45). However, this number was pre‐determined with sample size calculations to ensure accurate comparison between groups. Image analysis was also completed by an investigator blinded to study affiliation to ensure precise glucose uptake values. Therefore, despite a smaller sample size, we see that our data accurately measures and compares inflammation between groups. Furthermore, as with any human subject study, self‐reporting is not entirely reliable (Gorber et al., 2009). Individual behavior data including e‐cigarette use or smoking frequency, daily alcohol intake, exercise frequency, and prescription medication usage was all self‐reported. Although not all behaviors listed could be quantified nor verified, we ensured an accurate measure of habitual use through the analysis of cotinine in blood samples on the day of the study (Raja et al., 2016). Lastly, plasma markers of inflammation and oxidative stress were not measured in this study. However, in prior studies using the highly sensitive methodology including flow cytometry with fluorescent probes in otherwise healthy young people, we have previously reported differences in oxidative stress and inflammation among the three cohorts (Gupta et al., 2021; Kelesidis et al., 2020, 2021a, 2021b). Although, in this report using PET/CT, we did not find differences among nonsmokers, e‐cigarette users, and tobacco users in inflammation in the splenocardiac axis, we feel it is best for this investigation to be replicated by other investigators.

## 7 CONCLUSIONS

In summary, there was moderate to strong correlation between metabolic activity in all regions of interest, including: the amygdala, bone marrow, spleen, and aorta, but not control tissue, skeletal muscle. Our results further underscore the role of the sympathetic nervous system, as activated by the amygdala, in provoking inflammatory monocyte proliferation and instigating atherosclerotic development. Although we hypothesized that differential chronic exposure to e‐cigarettes or tobacco cigarettes would lead inflammatory differences between groups, inflammation in each target tissue did not vary significantly between nonsmokers, and light e‐cigarette users, and light smokers.

## AUTHOR CONTRIBUTIONS

Isabelle Ruedisueli, BS: Participant recruitment, data management, drafting the manuscript. Sara Arastoo, MD: Participant recruitment, conduct of study, data management, editing and approving the final manuscript. Pawan K. Gupta, MD: Design of nuclear medicine protocol, analysis of all PET scans, editing and approving the final manuscript. Jeffrey Gornbein, Dr.PH: Statistical analysis, editing and approving the final manuscript. Holly R. Middlekauff, MD: Study design, funding, conduct of study, data interpretation, editing and approving the final manuscript.

## FUNDING INFORMATION

This work was supported by Tobacco Related Diseases Research Program (28IR‐0065), the Graham Foundation, and the Sandler Foundation.

## CONFLICT OF INTEREST

None. The authors have no conflicts of interest or disclosures.
